# Modulatory effects of ayahuasca on personality structure in a traditional framework

**DOI:** 10.1007/s00213-020-05601-0

**Published:** 2020-07-23

**Authors:** Nige Netzband, Simon Ruffell, S. Linton, W. F. Tsang, T. Wolff

**Affiliations:** 1 University of West of England, Bristol, UK; 2 South London and the Maudsley, London, UK

**Keywords:** Ayahuasca, Dimethyltryptamine, Personality, Mystical/peak experience, Psychedelic, Entheogen, Psychopharmacology

## Abstract

**Abstract:**

Ayahuasca is a psychoactive plant brew containing dimethyltryptamine (DMT) and monoamine oxidase inhibitors (MAOIs). It originates from the Amazon basin, where it is used primarily for ceremonial purposes. Ayahuasca tourists are now entering certain communities seeking alternative physical or psychological healing, as well as spiritual growth.

**Rationale:**

Recent evidence has shown that the similar acting psychedelic compound, psilocybin, facilitated long-term increases in trait openness following a single administration.

**Objectives:**

This paper assesses the impact of ayahuasca on personality in a traditional framework catering for ayahuasca tourists.

**Method:**

Within a mixed design, we examined the effect of ayahuasca on participants’ personality (measured by the NEO Personality Inventory 3 questionnaire) across time (pre- to post-ayahuasca administration, and 6-month follow-up), relative to a comparison group (who did not ingest ayahuasca).

**Results:**

The results demonstrated significant increases in agreeableness pre- and post-ayahuasca administration and significant reductions in neuroticism in 24 participants, relative to the comparison group. Both of these changes were sustained at 6-month follow-up, and trait level increases were also observed in openness at this stage. Additionally, greater perceived mystical experience (measured using the Mystical Experience Questionnaire 30) was associated with increased reductions in neuroticism.

**Conclusions:**

These findings, which indicate a positive mediating effect of ayahuasca on personality, support the growing literature suggesting potential therapeutic avenues for serotonergic psychedelics.

## Introduction

Anthropological data suggests that psychoactive compounds have been used throughout history and are still used to this day by various traditional communities. Some examples include the Huichol peoples of northern Mexico, using peyote cactus (mescaline) (Schaefer 2006); the Mazatec use of psilocybin mushrooms (Metzner 2005); the Bantu tribes of Gabon who use iboga; and a number of communities within the Amazonas which use ayahuasca. These traditions appear to share similarities, despite the vastness of their geographical separation (Luna 2011), the most obvious being the type of compound used, often existing in different species of plant or fungi; similarities in ritual; shape of ceremonial buildings; the use of music and perfumes; and often resemblances in spiritual themes (Winkelman 2013). These features appear to have evolved independently, with examples on entirely different continents (Clottes and Lewis-Williams 1998). From this, it can be deduced that these practices possess some form of therapeutic efficacy, given that the same conclusions appear to have been reached. Scientists have begun looking at these practices in order to investigate their potential applicability in medicine (Sessa 2012).

Ayahuasca is an Amazonian plant brew mix in the entheogenic (i.e. spirituality inducing) bracket of psychedelics (Tupper 2009). The term ayahuasca originates from the Quechua words ‘Aya’—meaning soul or spirit—and ‘Waska’—meaning rope or vine (Santos et al. 2007). These names relate to one of the primary ingredients, the *Banisteriopsis caapi* vine, also independently referred to as ayahuasca. Currently, the most widely used brew contains *Banisteriopsis caapi* alongside one other dimethyltryptamine (DMT)–containing plant, usually *Psychotria viridis* (Rivier and Lindgren 1972).

Ayahuasca’s psychoactive effects are largely a result of DMT, which remains orally active due to monoamine oxidase inhibitors (MAOIs) present in *Banisteriopsis caapi* (McKenna 2004). Monoamine oxidase (MAO) is an endogenous enzyme which ordinarily breaks down DMT when orally ingested (McKenna et al. 1984), inhibiting its psychoactive properties. Combining the two plants allows DMT to be slowly absorbed in the digestive tract, triggering an experience lasting between 4 and 6 h (Riba et al. 2003), frequently encompassing powerful shifts in perception (Shanon 2002). In addition, users can experience purgative effects (Gershon 2004) such as vomiting (Tafur 2017).

The primary activation site for DMT is the 5-hydroxy-tryptamine (5-HT2A) receptor (Aghajanian and Marek 1999), similar to that of other serotonergic psychedelics with DMT-like chemical structures, such as lysergic acid diethylamide (LSD) and psilocybin (Nichols 2016). The 5-HT2A receptor has been linked to conditions such as depression (Celada et al. 2004), suggesting that psychedelics may hold therapeutic value in psychiatric disorders due to their prominent affinity here. Evidence suggests that these 5-HT2A agonists can decrease functional connectivity in the default mode network (DMN) (Carhart-Harris et al. 2016). This disruption in neural connectivity has been proposed to underlie subjective reports encompassing a loss of sense of self, ego-dissolution, often described as a transcendental state of awareness or mystical experience (Barrett and Griffiths 2017a).

A systematic review (dos Santos et al. 2016) assessing 28 publications on ayahuasca drew the following conclusions: acute ayahuasca administration was well tolerated (Fortunato et al. 2009); it was found to alter visual perceptions in participants (de Araujo et al. 2012), activate frontal and paralimbic regions (Riba et al. 2006), decrease DMN activity (Palhano-Fontes et al. 2015), and impair working memory but decrease stimulus-response interference (Bouso et al. 2013). Post-acute effects included improved planning and inhibitory control (Bouso et al. 2012), anti-depressive (Osório et al. 2015), and anti-addictive properties (Berlowitz et al. 2019; Fábregas et al. 2010; Thomas et al. 2013). Long-term ayahuasca use was associated with the increased cortical thickness of the anterior cingulate cortex and cortical thinning of the posterior cingulate cortex (Bouso et al. 2015).

Subacute and long-term ayahuasca use was not associated with increased psychopathology or cognitive deficits (Bouso et al. 2012) but was associated with enhanced mood and cognition (Bouso et al. 2012) and reduced impulsivity (Bouso and Riba 2014). Furthermore, several Brazilian studies have shown that a single dose of ayahuasca can have a rapid anti-depressant effect on patients suffering from recurrent depression (Osório et al. 2015; Palhano-Fontes et al. 2019; Sanches et al. 2016).

Animal studies indicate that the median lethal dose of DMT in humans would amount to 20 times more than that used in ceremonial ayahuasca practice (Gable 2007), and neither acute ayahuasca administration nor long-term consumption seems to be toxic to humans (dos Santos 2013). Use of the brew in religious ceremonies has a safety margin comparable to codeine, mescaline, or methadone (Gable 2007), with minimal risk of sustained psychological disturbance. Cardio-vascular risk has been found to be low (Riba et al. 2003), as has the addiction potential of the brew (Fábregas et al. 2010). In fact, no serious conditions have been established when consumed by healthy individuals (dos Santos 2013).

Despite evidence pointing to an acceptable safety profile for ayahuasca, there have nonetheless been cases observed where acute ingestion has been a contributing factor to psychotic manifestations (dos Santos et al. 2017; Tófoli 2011). For example, a number of such cases have been documented by churches utilizing ayahuasca, such as the União do Vegetal (UDV). However, it has been impossible to directly infer causality due to factors such as temporality (Tófoli 2011) and additional use of substances such as cannabis (dos Santos and Strassman 2008). It should also be noted that incidences of psychotic illnesses recorded within the UDV were estimated to be similar to those of the general population—around 1% (Gable 2007; Stilo and Murray 2010).

In a legal battle regarding ayahuasca use, the Supreme Court of the United States (Gonzales 2006) concluded that ‘many or most of these psychological problems were transient and resolved’, and in ‘a review of the case histories… either no truly psychotic incident was identified or no causal link to *hoasca* (ayahuasca) was found’. Overall, psychotic reactions to ayahuasca seem rare. When this phenomenon does occur, it is often associated with factors such as substance abuse, inappropriate, unsupervised settings, and predisposing psychological characteristics (dos Santos et al. 2017). Such cases further highlight the need for appropriate screening, setting, and support (Zinberg 1986).

Several studies have suggested that ayahuasca use can result in positive changes across different psychological and personality domains. In studying Santo Daime church members 1 to 2 weeks following their first-time ritualistic ayahuasca use, Barbosa et al. (2005) found significant reductions in minor psychiatric symptoms, as well as mood and behavioural changes related to greater assertiveness, serenity, and joy. In a sample of ayahuasca tourists which included one-time drinkers using the Personality Styles and Disorder Inventory (Kuhl and Kazén 2009), Kavenská and Simonová (2015) found significant increases in traits such as optimism, intuition, ambition, helpfulness, and charm. Authors noted that compared with controls, those using ayahuasca demonstrated more trustful, pleasant, empathic, and optimistic personality styles.

In exploring the effects of longer-term use, Barbosa assessed members of the UDV and Santo Daime churches for personality changes after drinking ayahuasca regularly for 6-months (Barbosa et al. 2009). Using the Temperament and Character Inventory (Cloninger et al. 1993), experimental groups showed significant reductions in reward dependence at 6-month follow-up which positively correlated with intensity of use. These findings supported the findings of an earlier, similar study (Grob et al. 1996). Barbosa and colleagues further found that Santo Daime members exhibited greater confidence and optimism at 6-month follow-up, which correlated positively with improvements in mental health and reduced minor psychiatric symptoms (Barbosa et al. 2009). This was supported by Bouso and his colleagues (Bouso et al. 2012) under both jungle and urban settings. Using the Big Five Inventory (BFI) (Goldberg 1990), significantly higher ratings of agreeableness and openness were observed in regular ayahuasca users (Barbosa et al. 2016).

When assessing psilocybin, another 5-HT2a agonist structurally-likened to DMT, positive changes have been identified. In a randomised controlled trial using the Neuroticism-Extraversion-Openness Personality Inventory (NEO-PI; Costa and McCrae 1992), MacLean and colleagues (MacLean et al. 2011) found significant increases in openness which persisted after 12 months. This finding positively correlated with levels of perceived mystical experience (measured using the Mystical Experience Questionnaire, MEQ). More recently, Erritzoe et al. (2018) conducted a similar study on individuals suffering from unipolar depression, observing significant increases in extraversion and openness, as well as significant reductions in Neuroticism, which also correlated positively with levels of perceived mystical experience. Such personality changes have been associated with reduced anxiety, depression, and alcohol and substance misuse (Kotov et al. 2010; Malouff et al. 2007; Ruiz et al. 2008).

Together, these studies provide foundational evidence suggesting that ayahuasca and similar compounds, like psilocybin, have the potential to bring about positive and sustainable changes to personality structures. Additionally, under the five-factor personality model (or Big Five), the literature suggests that around 30 is the age at which personality is seen to stabilise, with openness to experience considered to be the most substantially heritable trait (e.g. Costa and McCrae 1992; Jang et al. 1996; Terracciano et al. 2010). Since the aforementioned studies conducted under the Big Five model (i.e. those utilising the BFI and NEO-PI) involved participants mostly beyond age 30, it is further suggested that these compounds may influence sustainable change to personality traits previously thought to be largely inherited and stable.

In the current study, understood to be the first of its kind, we aimed to evaluate ayahuasca administration in a non-church-based traditional Amazonian setting investigating effect on personality, in both the short-term and six-month follow-up. Given its potential positive effects on personality and treatment implications of psychiatric disorders, coupled with the continued rise in ayahuasca tourism, this area of research holds great importance. Our study was conducted within an indigenous Shipibo community of the Peruvian Amazon. The Ayahuasca Foundation, affiliated with the Multidisciplinary Association for Psychedelic Studies (MAPS), allows for ‘ayahuasca tourists’ to participate in Shipibo-style ceremonies which closely resemble the traditional use of the plants within this area of the Amazon basin.

## Methodology

This field study was conducted using an observational repeated measures design.

### Location and retreat information

Data was collected at the Ayahuasca Foundation, an ayahuasca retreat and research centre within the Allpahuayo-Mishana National Reserve, approximately 20 miles from Iquitos. Each retreat lasted 12 days and included six ayahuasca ceremonies every other day. A range of other plants were used during the retreats, none of which is known to be directly psychoactive.

The administration of ayahuasca was conducted in the format of a non-religious ceremony, with its traditions rooted in Shipibo culture. These were led by a local curandero (shaman), alongside four to five facilitators trained by the foundation. Ayahuasca ceremonies generally began after sunset at around 20:00, lasting approximately 6 h. The circular wooden building where these occurred (i.e. the ‘maloka’) was set up with single mattresses evenly spaced out around the inside perimeter for each participant, with individual buckets for purging provided. Ceremonies were conducted in groups of around 10 to 12 participants. Throughout, participants remain silently on their mattresses in complete darkness, without making contact with one another. Toilet breaks were allowed, with assistance from facilitators if necessary. After the curandero presents each with the ayahuasca brew at the start, he and the facilitators would sing traditional medicine songs (‘icaros’) for the duration of the ceremony.

To ensure participants’ peace of mind on retreats, the foundation provided security measures such as locked rooms and over-night guards against potential threats from the jungle. A medical doctor accredited with primary qualifications was around in case medical assistance was needed, and aftercare remote professional counselling sessions were also offered following retreats to promote integration.

### Sample group

Twenty-four English-speaking individuals (15 males, 9 females, mean age = 37.6) participated. Twenty-one were either of white American, Canadian, British, or European ethnicity, with the remainder being Asian or Latin. All were vetted by the Ayahuasca Foundation staff for eligibility regarding physical and mental health. Half of the participants had either historical or ongoing diagnosed psychiatric conditions (including depression, anxiety, and post-traumatic stress disorder), who met the Ayahuasca Foundation’s inclusion criteria. Five had chronic on-going physical ailments. Nine had suffered previous physical ailments which had resolved. Four had no diagnostic history of either physical or psychiatric conditions. All participants received full-time education until the age of 16. Nine of these further received qualifications at A-level or college level. Ten continued further on to the university and postgraduate level. Half reported previously having experienced drinking ayahuasca prior to the retreat.

#### Exclusion criteria

The Ayahuasca Foundation excluded individuals with current or historic psychosis-related conditions, such as bipolar depression or schizophrenia. All individuals are encouraged to abstain from licit and illicit substances (including prescription medication) for a period of 2 weeks prior to retreats. Medical records were not checked, and therefore, participants were accepted based on their subjective account of their medical history*.*

### Comparison group

Comparison group participants were English-speaking individuals on holiday in Peru who were initially approached on the premise that they had no previous experiences with ayahuasca reported. In total, 65 data sets were obtained, with 24 subjects (11 males, 13 females, mean age = 32.6 years) being selected for a matched comparison group. An independent samples *t* test revealed no significant difference between the comparison and sample groups on age, *t* (46) = 1.43, *p* = .160. Chi-squared tests also revealed no significance difference between the two groups on gender, *X*^2^ (1) = 0.75, *p* = .386; education, *X*^2^ (2) = 3.11, *p* = .211; whether they had historic or current psychiatric diagnoses, *X*^2^ (1) = 0.09, *p* = .768; and physical health issues, *X*^2^ (1) = 0.33, *p* = .564.

### Measures

The *NEO-PI3* (NEO henceforth) (Costa Jr and McCrae 2008) was selected as a robust personality measurement with high overall validity and reliability. The questionnaire identifies five primary personality domains, each of which has six sub-facets: openness to experience, conscientiousness, extraversion, agreeableness, and neuroticism.

The Mystical Experience Questionnaire (MEQ30; Barrett and Griffiths 2017b) was used to assess levels of perceived mystical experience.

### Procedure

Written consent and demographic information were obtained the day before the first ayahuasca ceremony, followed by a time one baseline personality measurement using the NEO. Time two NEO scores were recorded at the end of the retreat following six ceremonies. In the post-phase, the MEQ was also administered.

The 6-month follow-up NEO scores were obtained electronically via email. This also included a follow-up questionnaire assessing the potential long-term impact of the retreat in terms of behavioural, physical, and psychological changes.

Over the 12-day retreat, semi-structured interviews were conducted. These qualitative findings are presented in a separate publication (Wolff et al. 2019).

### Ethics

This study has been approved by the institutional ethics committee and was independently assessed by the British Psychological Society (BPS).

## Data analysis

We first assessed whether the ayahuasca sessions led to changes in personality from baseline to post-test, using a mixed ANOVA, with time (baseline, post-test) and personality (neuroticism, extraversion, conscientiousness, agreeableness, openness to experience) as the within-participants variables, and group (active vs. comparison) as the between-subjects variable (see Table 1 for descriptive statistics). To correct for multiple comparisons, the Benjamini-Hochberg procedure (Benjamini and Hochberg 1995) was applied for all follow-up pairwise comparisons with a false discovery rate (FDR) of 0.1. The Greenhouse-Geisser correction was used in instances where the assumption of sphericity was violated (Mauchly’s *W* < .05). After confirming that neuroticism reduced significantly, we used a bivariate Spearman’s rank-order correlation to assess the relationship between neuroticism and the mystical experience reported by participants.

**Table 1:** NEO Personality scores for the active and comparison group

	Baseline		Post-test		6-month follow-up	
	(M, SD)	Range	(M, SD)	Range	(M, SD)	Range
Active group						
Neuroticism	90.08 (27.68)	48–155	73.00 (30.56)	29–136	75.90 (23.69)	31–116
Conscientiousness	113.79 (21.82)	75–154	118.21 (23.46)	62–156	118.00 (17.49)	80–155
Extraversion	113.21 (17.35)	83–150	117.79 (22.72)	74–159	120.57 (19.40)	89–156
Agreeableness	114.75 (22.99)	74–152	124.88 (22.47)	82–161	119.33 (19.57)	82–158
Openness to experience	128.58 (18.06)	84–159	135.50 (23.06)	67–166	135.48 (17.99)	101–167
Comparison group						
Neuroticism	96.79 (24.54)	58–143	93.42 (24.07)	61–137	109.83 (15.01)	70–135
Conscientiousness	108.50 (15.58)	84–141	106.83 (15.59)	77–136	118.83 (7.56)	102–131
Extraversion	121.13 (21.70)	84–167	119.88 (19.10)	89–150	117.46 (10.45)	92–134
Agreeableness	131.00 (19.49)	90–169	126.21 (22.53)	65–169	123.38 (6.97)	110–139
Openness to experience	132.67 (16.83)	103–165	131.08 (20.42)	75–161	117.08 (8.25)	95–130

## Results

### Personality changes from baseline to post-treatment

**Fig. 1:**
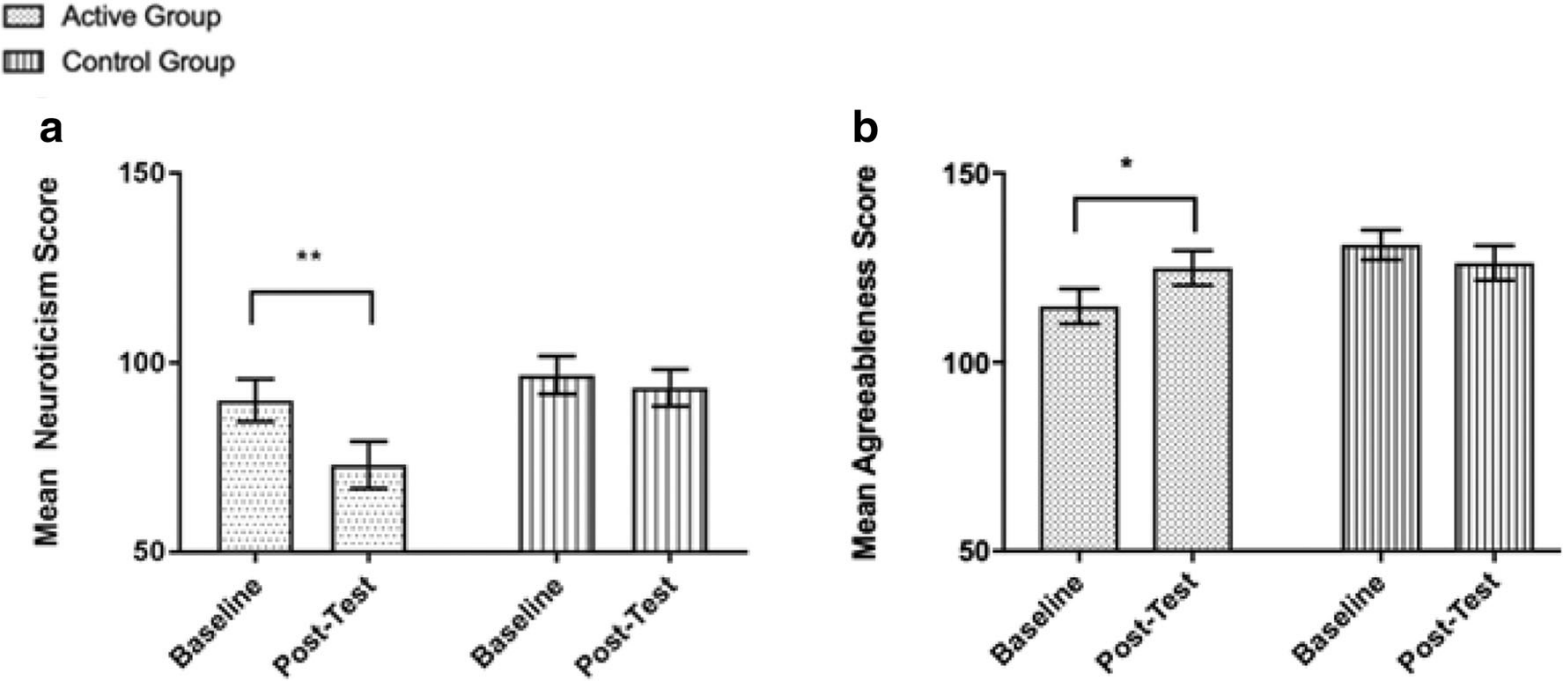
Significant reduction in neuroticism (**a**) and increase in agreeableness (**b**) observed in the active group from baseline to post-test, compared with the comparison group. Asterisk indicates *p* < .05, double asterisk indicates *p* < .001. Bars represent the standard error of the mean (SEM)

Analysis observed a significant reduction in neuroticism scores in the active group at post-test, as indicated by a significant interaction between time, personality, and group (Pillai’s trace = 0.24, *F*(2.03, 93.44) = 5.50, *η*_p_^2^ = .11, *p* = .005). Pairwise comparisons revealed a significant reduction in Neuroticism scores from baseline measures to post-test in the active group (Mdiff = 17.08, 95% CI (10.12, 24.05), *p* < .001), *d* = 0.59, not the control group (Mdiff = 3.38, 95% CI (− 3.59, 10.34), *p* = .335, *d* = 0.14). Given that there were non-significant differences in baseline neuroticism scores between the active and comparison groups, Mdiff = 6.71, 95% CI (− 8.49, 21.91), *p* = .379, *d* = 0.26, our results suggest a significant reduction in neuroticism in the active group following the ayahuasca sessions. Pairwise comparisons also revealed a significant increase in agreeableness scores from baseline measures to post-test in the active group (Mdiff = 10.13, 95% CI (2.34, 17.91), *p* = .012), *d* = 0.45, not the comparison group (Mdiff = 4.79, 95% CI (− 3.00, 12.58), *p* = .222) (Fig. 1). Consistent with our original hypothesis, there was a trend towards a significant increase in openness scores from baseline to post-test in the active group (Mdiff = 6.92, 95% CI (0.32, 13.51), *p* = .040); however, this test did not survive the correction for multiple comparisons.

### Relationship between neuroticism, agreeableness, and mystical experience

**Fig. 2:**
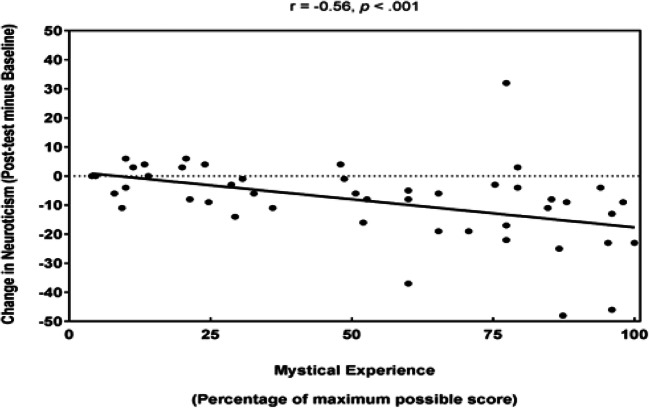
Graph to show the change in neuroticism scores in both the active and comparison group as a function of mystical experience, including a line of best fit

To assess whether the reduction in neuroticism and increase in agreeableness observed in the active group were associated with the degree of mystical experience reported by participants, we conducted a Spearman’s rank-order correlation between neuroticism and agreeableness change scores from baseline to post-test in both the active and comparison groups and their MEQ scores. This analysis revealed a medium significant negative correlation between neuroticism change and MEQ scores, *r*_s_(48) = − .56, *p* < .001 (i.e. those who reported a greater degree of mysticism also experienced greater reductions in neuroticism) (Fig. 2). We can also report a non-significant correlation between baseline neuroticism and MEQ scores, *r*_s_(48) = .02, *p* = .883, which suggests that it is the change in neuroticism from baseline to post-test that is driving the significant association with MEQ scores. In contrast, there was a non-significant correlation between agreeableness change and MEQ scores, *r*(48) = 0.18, *p* = .211.

### Personality changes from post-treatment to follow-up

Out of the 24 participants in the active group, three did not provide NEO scores at the 6-month follow-up. Therefore, the 6-month follow-up analyses were conducted with 21 participants in the active group (12 males, mean age = 34.2 years) and 24 participants in the comparison group.

**Fig. 3:**
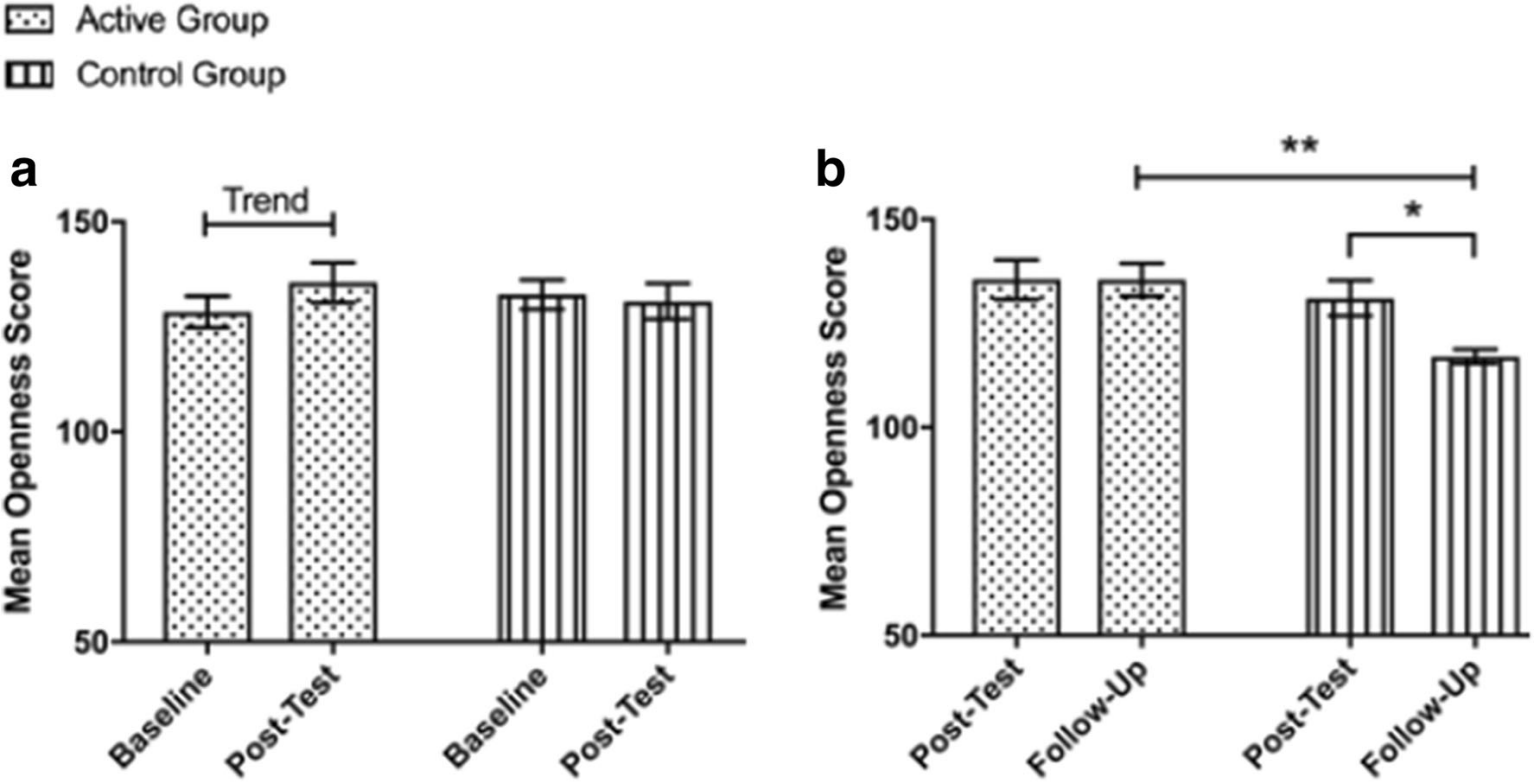
Trend level increases in openness which were observed in the active group from baseline to post-test (A), and the significant decrease in openness which was observed in the comparison group from post-test to six-month follow-up. At 6-month follow-up participants in the active group had significantly greater openness scores than the comparison group. Asterisk indicates *p* < .005, double asterisk indicates *p* < .001. Bars represent SEM

**Fig. 4:**
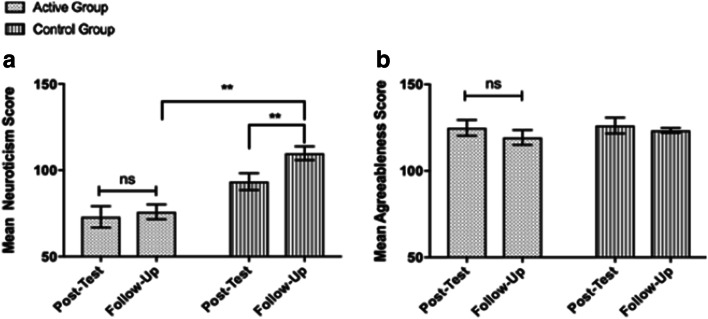
Significant reduction in neuroticism (**a**) and increase in agreeableness scores (**b**) observed in the active group at post-test remained stable at 6-month follow-up and were significantly reduced in comparison with the comparison group at follow-up. In contrast, we observed a significant increase in neuroticism scores in the comparison group from post-test to 6-month follow-up. Double asterisk indicates *p* < .001. Bars represent SEM

Our data suggests the significant reduction in neuroticism scores observed in the active group remains stable at 6-month follow-up, relative to participants in the comparison group, as indicated by a significant interaction between time, personality, and group, Pillai’s trace = 0.22, *F*(3.17, 136.24) = 4.32, *p* = .005, *η*_p_^2^ = .09 (Fig. 3). Pairwise comparisons revealed that the reduction in neuroticism scores observed in the active group at post-test remained was stable at the 6-month follow-up assessment (*M* = 75.91, SEM = 4.26), Mdiff = 2.29, 95% CI (− 5.16, 9.73), *p* = .539, *d* = 0.08, and remained significantly lower than those observed in the comparison group (*M* = 109.83, SEM = 3.99), Mdiff = 33.93, 95% CI (22.16, 45.70), *p* < .001, *d* = 1.71. In addition, the short-term increase in agreeableness that was observed in the active group was maintained at 6-month follow-up (*M* = 119.33, SEM = 3.12), Mdiff = 6.14, 95% CI (− 2.33, 14.62), *p* = .151, *d* = 0.26 (Fig. 4). Lastly, at 6-month follow-up, we also observed significantly greater openness to experience scores in the active group (*M* = 135.48, SEM = 2.98) compared with the control group (*M* = 117.08, SEM = 2.79), Mdiff = 18.39, 95% CI (10.15, 26.63), *p* < .001, *d* = 2.20.

## Discussion

The current study shows that a 12-day ayahuasca retreat in a traditional framework adapted for ayahuasca tourists led to significant reductions in neuroticism, which remained stable at 6-month follow-up. Additionally, MEQ scores were found to correlate with reductions observed in neuroticism. These findings are in line with previous research (Erritzoe et al. 2018). Increases in agreeableness were observed from baseline to post-test in both the short and long term, supporting findings by Barbosa et al. (2016). Trait level increases in openness were also observed, which further increased between the post-test and 6-month follow-up, a finding which again is partially supportive of previous findings (Barbosa et al. 2016; Maclean et al. 2011).

Results show that reductions in neuroticism were more pronounced than we anticipated. In addition, levels of neuroticism in the comparison group were found to unexpectedly increase at 6-month follow-up. We speculate that as the majority of participants had returned home from travel by this point, those increases in neuroticism may represent changes in a frame for the personality requirements of the individual, i.e. they became more neurotic upon return to their non-travelling lives. These findings are particularly interesting as personality is often seen to be stable by age 30, unless a significant life event occurs (Costa and McCrae 1992). It is suggested that personality may in fact not be as rigid as advocated by Costa and McRae.

Neuroimaging data has shown that serotonergic psychedelics may amplify the mechanism of neuroplasticity (Carhart-Harris et al. 2012), the brain’s ability to be flexible and form new pathways (Costandi 2016). Ayahuasca may be unique in its ability to induce such processes. A study conducted by Morales-Garcia et al. (2017) demonstrated evidence that the alkaloids present in *B. caapi* stimulate neurogenesis in human hippocampal tissue in vitro. These results, when combined with the implications of functional suppression of the DMN (Palhano-Fontes et al. 2015) and increases in neuroplasticity induced by DMT (dos Santos and Hallak 2019), suggest that ayahuasca as a concoction may possess serious power for bringing about neurological alterations in the brain. It must be noted however that the findings by Morales-Garcia have not yet been replicated in vivo*.* Direct transferability into humans at this point is therefore solely speculative. Regardless, these findings have opened up fascinating research avenues for conditions such as neurodegenerative disorders, or even age-related cognitive deficits.

A recent study (Erritzoe et al. 2018) showed significant reductions in neuroticism in patients suffering from treatment-resistant depression. However, the inclusion criteria for the above study are much more restrictive than those in the current paper (diagnosed patients vs. self-selection); the validity of comparing these findings is therefore questionable. Future studies should aim to examine the differences in outcomes between healthy individuals and those with diagnosed conditions with a basis in neurosis (e.g. affective disorders), when receiving psychedelic therapy.

In contrast to the findings observed by Maclean et al. (2011), we observed no significant effect on levels of openness, only observing trait level increases. However, the sample group in the current study reported higher levels of openness relative to the general population, where openness was already at or close to ceiling level. High levels of openness may reflect their natural inclination to seek out new experiences, or potentially that they may have already been primed to be more open than they would be in their day to day lives.

### Therapeutic implications

The results in this study are consistent with the growing body of data suggesting psychedelics have therapeutic implications. High levels of neuroticism are associated with a range of psychiatric conditions, such as anxiety, depression, and obsessive-compulsive disorder. The therapeutic applications of increased levels of agreeableness are however less obvious. Agreeableness can be defined as an individual’s pro-social behaviour relating to characteristics such as altruism, empathy, and cooperativeness (Caspi et al. 2005). It has previously been suggested that agreeableness may be a reliable predictor of substance misuse (Turiano et al. 2012); however, this is not the case for all substances. It has been found to be protective against the problematic use of cannabis (Terracciano et al. 2008), alcohol (Turiano et al. 2012), and polydrug use (Lackner et al. 2013). Agreeableness, along with neuroticism, is associated with traits of anger proneness (Caspi et al. 2005). Neuroticism refers to the way in which aggression is experienced through angry emotions, whereas agreeableness is associated with poor control expressed through aggression (Martin et al. 2000).

Much emphasis has been placed on the psychotherapeutic frameworks surrounding psychedelic sessions in determining therapeutic outcomes, a case of optimising set and setting (Erritzoe et al. 2018; Hartogsohn 2016; Mithoefer et al. 2018). Most of the psychedelics showing promise in publications appear to be 5-HT2A/C agonists. This suggests a potential link between this receptor site and neurotic behaviour. Many researchers (including MacLean et al. 2012) suggest this receptor is responsible for inducing peak, or mystical, states. It is currently unclear whether direct agonism of the receptor, without experiential effects, may bring about reductions in neuroticism and increases in agreeableness, or indeed if this is possible. It is also unclear whether or not changes in psychological state are the primary mediator, or the degree to which this is affected by set and setting. Repeating this study in a variety of settings, such as neo-shamanic or clinical, would aid in providing greater insight.

Ayahuasca has been found to elicit changes in personality structure in a number of studies in different settings (Barbosa et al. 2009; Grob et al. 1996; Kavenská and Simonová 2015). Many of the changes recorded suggest therapeutic implications for affective disorders, often associated with high levels of neuroticism (Duggan et al. 1995). In addition, increasing agreeableness and decreasing neuroticism on the NEO may benefit those suffering from cluster C personality disorders, such as obsessive-compulsive personality disorder. This study demonstrated a positive relationship between the extent of a perceived mystical experience and changes in personality structure. This finding is consistent with recent work that suggests the therapeutic relevance of mystical states in psychedelic therapy (e.g. MacLean et al. 2011). It is important to continue studying traditional communities and the efficacy of their practices while also being mindful of the limitations to adapting them to Western frameworks.

### Ethical issues surrounding ‘ayahuasca tourism’

It should be noted that the recent increase in ‘ayahuasca tourism’ is not without risks. Individuals claiming to be ‘neo-shamans’ who have not undergone the extensive training typically required to bear such a title is a prime example of this (De Rios 2009). These happenings raise a vast array of concerns, such as the safe preparation of brews, potential contraindications, financial and other types of exploitation, and potential cases of sexual abuse by predatory folk masquerading as healers (Prayag et al. 2016). In Peru, there is practically no regulation or vetting for practitioners. It is therefore up to the individual to ensure the expertise and intentions of people they choose to drink with. There are also issues surrounding cultural appropriation as the use of ayahuasca grows. This is principally due to its status as a sacrament within certain communities (Tupper 2009). Although it is beyond the scope of this paper to discuss, readers should be aware that so-called ‘retreat centres’ based in locations such as the Amazon have come under scrutiny, with opposition to the potential financial gain some non-indigenous individuals stand to make from ayahuasca tourism and other schemes surrounding plant medicines.

### Limitations

Some limitations should be considered when interpreting our findings. Firstly, this was a field experiment, which naturally gave rise to a number of confounding variables researchers were unable to control for. Readers should therefore exercise caution when inferring causality and generalising findings outside the setting outlined in this study. For instance, researchers were limited in access to equipment which could have established neurobiological and pharmacokinetic correlates to findings, via plasma and other appropriate biological data, as well as potential epigenetic markers. As several other non-psychoactive plants and treatments were used in conjunction with ayahuasca, it is impossible at this stage to quantify the potential impact of these variables on our outcome measures. This was principally a resource issue as a result of being independently funded.

Although participants were asked to abstain from psychoactive substances between the retreat and 6-month follow-up, it was impossible to ascertain the extent to which this was adhered to. Namely, the current study format did not allow for us to stringently control, test, or exclude anyone engaging in psychoactive substance use. According to the self-reported data at follow-up, drug use appears to have decreased overall following ayahuasca sessions within our participants. Future consideration should be put into longitudinal designs which allow the controlling of restricted, or at least minimised, psychoactive substance use.

Similarly, other potentially impactful lifestyle variables were not monitored. This largely relates to integration or psychological support individuals may have engaged in which may have affected the validity of long-term findings.

Due to the nature of the retreat, no set doses of ayahuasca were specified. Instead, each dose was based upon both the curandero’s recommendation and the individual’s will on the day of the ceremony. While this may mean scientific standardisation is unmet, insistence on consistent dosing would have deviated from the traditional framework on which this culture-specific observational study was based. Furthermore, due to the study being an independent self-funded project, funds/resources were insufficient for constituent analysis of ayahuasca samples (e.g. use of high-performance liquid chromatography).

It is likely that the results obtained are subject to self-selection bias, as individuals who opted into the study have made time/financial sacrifices to attend the retreat. Such would naturally elicit some degree of expectations which may well affect outcomes.

Finally, the observational nature of the study also gave rise to potential safeguarding concerns. While there was a medical doctor on-site and evidence suggests an acceptable safety profile for ayahuasca use, participants’ medical records were not obtained to confirm self-reported medical and psychiatric histories. It was therefore difficult to adequately safeguard against unreported conditions having potentially unknown negative interactions with ayahuasca use. Fortunately, no medical assistance was required for the current sample, but this nonetheless raises ethical considerations for ayahuasca retreats more broadly. Greater control of inclusion/exclusion criteria in future studies may potentially cater for both safeguarding considerations and external validity of findings.

### Summary

Increasingly, traditional practices are being incorporated into modern therapeutic frameworks. It is feasible that other treatments, such as variations on the South American shamanic practices, may one day be conducted in clinical settings, as cultures continue to converge.

As interest in psychedelics grows both in the scientific community and the public, the efficacy and safety of these compounds require further investigation using rigorous research design, under state-sanctioned regulation, in a range of frameworks. In doing this, we can fully investigate therapeutic avenues for these ‘novel treatments’, particularly as these compounds appear to possess what appears to be powerful potential for aiding the human condition on an every-day level.

The current study goes some way in validating the effectiveness of adapted traditional ayahuasca retreats on ayahuasca tourists, by providing personality data which may imply therapeutic avenues.
